# Medical student mistreatment: understanding ‘public humiliation’

**DOI:** 10.1080/10872981.2019.1615367

**Published:** 2019-05-08

**Authors:** Jesse D. Markman, Thomas M. Soeprono, Heidi L. Combs, Ellen M. Cosgrove

**Affiliations:** a Department of Psychiatry and Behavioral Sciences, University of Washington, Seattle, WA, USA; b Department of Medicine, University of Nevada, Las Vegas, USA

**Keywords:** Medical student mistreatment, public humiliation, student embarrassment, learning environment

## Abstract

**Introduction**: Mistreatment in medical school is an enduring problem in medical education. Little is known about the concept of ‘public humiliation,’ one of the most common forms of mistreatment as identified on the AAMC Graduation Questionnaire. The objective of this study was to further investigate ‘public humiliation’ and to understand the underpinnings and realities of ‘public humiliation’ in medical education.

**Method**: Focus groups of medical students on clinical rotation at the University of Washington School of Medicine were conducted over one and a half years. Qualitative analysis of responses identified emergent themes.

**Results**: Study results included responses from 28 third year and one fourth-year medical student obtained over five different focus groups. Participants defined the term ‘public humiliation’ as negatively, purposefully induced embarrassment. Risk factors for the experience of public humiliation in educational settings were found to include the perceived intent and tone of the teacher, as well as situations being ‘public’ to patients and taking place during a medical or surgical procedure. Socratic teaching or ‘pimping’ was not found to be a risk factor as long as learners were properly oriented to the teaching practice.

**Discussion**: This study investigated and defined ‘public humiliation’ in the setting of medical student mistreatment. More subtle forms of mistreatment, like public humiliation, may be amenable to interventions focused on teaching educators about the importance of orientation and clear communication of intent during the teaching process.

## Introduction

Mistreatment in medical school is not a new phenomenon. Psychological studies began reporting mistreatment as early as the 1960s and one study reported rates of mistreatment as high as 96% in 1991 [1,2]. Unfortunately, mistreatment has become an ingrained part of medical education that leads to medical student burnout, and poor mental health [3–12]. While very overt forms of mistreatment and abuse (e.g., sexual harassment and physical abuse) are better understood, more subtle or subjective forms, such as ‘public humiliation’ are less well conceptualized and understood in the academic literature. Medical education and other areas of higher education have struggled with how Socratic teaching techniques can be used effectively in the education of learners. These techniques have both been blamed for the humiliation of learners and argued as misunderstood, but effective teaching techniques [13,14]. Given ‘public humiliation’ was the most commonly perceived form of mistreatment in the Association of American Medical Colleges (AAMC) Medical School Graduation Questionnaire from 2000 to 2012, understanding ‘what is public humiliation’ and ‘what causes a situation to be publicly humiliating’ are important to determining how best to improve the medical student experience [15,16]. From 2013 to 2015, our group designed and implemented a research protocol utilizing focus groups of medical students to explore these exact questions and better understand medical student experiences of and definition of ‘public humiliation’ in the setting of clinical training.

## Methods

We conducted a qualitative, narrative study of medical student experiences and perceptions around the subject of ‘public humiliation’ using a series of student focus groups. Focus groups utilize a semi-structured set of questions to elicit responses from a group of participants. The discussion that results from the questions can vary as the group composition drives different responses, which may be elaborated upon by the different participants. This provides an opportunity for response variation among groups and interaction between focus group participants (i.e., participants seeking clarification from one another about the statements made in response to questions) leading to a level of response definition and clarification within groups that individual surveys cannot attain. We developed the question guide used in our groups from our review of relevant literature and the study investigators own experiences on the topic. The questions used are listed in Table 1. The Institutional Review Board of the University of Washington reviewed and approved the study.

**Table 1.** Focus group questions.

What clerkships have each of you done and where?
Were those clerkships positive or negative experiences?
Think about your clerkships. How were you treated?
Did public humiliation have anything to do with this?
What is the difference (if any) between: embarrassment, public humiliation, mistreatment, and abuse?
Where does ‘pimping’ fit in?
How do these situations vary between the locations and sites that clerkships are at?
How do these situations vary between specialties?
How does the Dean’s Office play a role in this problem?
What other factors may play a role in one’s perception of public humiliation?
What determines whether or not you report these incidences?
What needs to change to make this better?

## Participants

Study participants were limited to third and fourth-year medical students for the purpose of gathering data from those students who are closest to their clinical training experiences in medical school. Initially, third and fourth-year medical students at the University of Washington were recruited using a series of email advertisements. Recruitment proved difficult and participation was limited. Only one focus group was completed despite heavy email advertising. As a result, the study protocol was altered to accommodate direct, in-person recruitment of third-year medical students during orientation for their core psychiatry clerkship. Recruitment was completed by an attending psychiatrist involved in the study, but with no supervisory relationship, or clinical interaction with any of the students. While third-year medical students about to start their psychiatry rotation were directly recruited, all third and fourth-year medical students were eligible to participate, regardless of their current clinical rotation. Written, informed consent was obtained prior to participation. In total, 29 students (28 third year and one fourth-year medical student) were recruited to participate over five different focus groups.

## Data collection

Focus groups took place from the spring of 2013 to the winter of 2015. Prior to the beginning of each focus group, participants completed a short, quantitative survey consisting of questions related to medical student mistreatment as asked on the 2012 AAMC Medical School Graduation Questionnaire. Participants were then engaged in a qualitative focus group with study investigators acting as focus group moderator and co-moderator. All groups were audio recorded and lasted between 60 and 90 min. A semi-structured focus group guide was utilized with structured, initial questions, and optional follow-up questions, investigating medical student mistreatment and perceptions about ‘public humiliation.’ Participants self-selected identification numbers for self-reference and identification during discussions to increase statement anonymity. The audio-recordings of each focus group were transcribed verbatim and examined for any information that could be utilized to identify study participants or individuals within the University community. Any identifying information, which was extremely limited, was redacted from transcripts prior to analysis. Examination and subsequent redaction were first completed by transcription staff with no affiliation with the project or the school of medicine. The lead investigator then completed a second review to ensure that redaction had been complete.

## Analysis

Although data collection was completed at the level of the group given the focus group design, individual participant statements, within each focus group, were considered to be the data points for analysis. We conducted a conventional content analysis to direct the coding process. In a conventional content analysis, qualitative codes are derived directly from the data and not guided by a pre-existing theory as in a directed content analysis [17]. In addition, the analysis does not involve the counting or comparison of keywords or codes in the same way as in a summative content analysis [17]. We used the constant comparative technique from grounded theory to guide the coding process in examining the narrative for interconnecting categories of information [18]. We did not, however, engage the Grounded Theory as a methodology as we did not seek to establish theoretical constructs for this study. Open, thematic coding was independently completed by J.M. and T.S. to ensure interrater reliability. Each coder completed a second level of coding for synthesis. These results were compared and any discrepancies that existed were reviewed and resolved, generating the final themes from the analysis. The quantitative survey results were compiled and quantitatively analyzed to compare the identification of experienced or observed mistreatment among the study participants with national-level data from the AAMC questionnaire. We made use of a number of different strategies to enhance the credibility of our qualitative analysis and results as recommended by Patton [19]. The use of multiple focus groups provided multiple, separate data sources for analysis, which lent to the credibility of results. In addition, the use of the constant comparative technique in the content analysis enhanced credibility. Finally, the use of two, separate coders to independently complete open and second level coding supported the credibility of results.

## Results

Five different focus groups were conducted from the spring of 2013 to the winter of 2015. In total, 28 third-year medical students and one fourth-year medical student participated in the focus groups. Average group size was 5.8 (SD 2) students per group. Each focus group produced rich discussions on multiple themes. The previous clinical training experiences of the third year medical students participating in the groups varied. The thematic coding produced by each focus group was remarkably consistent across groups. No single group contained major themes that were not present in all other groups, and subsequent groups were not adding new information to our understanding of our research questions. We felt confident that our results had reached saturation, as defined by Creswell [20]. Given saturation had been reached, no further focus groups were held. Major findings from our qualitative analysis are reviewed in the subsections below with representative quotes for the noted themes. Additional, representative quotes linked to their coded themes can be found in Table 2. The example quotations are linked to the question(s), which generated those data during the focus groups.

**Table 2.** Focus group quotations.

Question	Example Quotations	Major Themes
Think about your clerkships, how were you treated? Did public humiliation have anything to do with this?	I would see her all the time with my badge, so I knew that she could see my name. I didn’t expect her to memorize my name, but knew she could see it. And she called me Med Student Number One. She would just say, ‘Med Student Number One, go grab this for me.’ He made fun of me for fumbling with equipment, or whatever, around patients, and that was probably the thing that I thought was the most obviously abusive, was like really just making fun of me. … and he’s saying things like, ‘Do you know how ridiculous you sound?’ and things like that, like yelling. And I could see the nurses kind of looking bad, like feeling bad for me.	Abuse – intentional cause of harm Negative treatment in front of patients and/or other staff – risk for humiliation Being disrespected or lack of respect
What is the difference between: embarrassment, public humiliation, mistreatment, and abuse?	… it’s all about how your attending responds to when you get something wrong. And it’s like, I don’t care if I get something wrong. I’m wrong all the time. But if they make me feel like I’m an idiot for getting something wrong or if they make me feel like ‘Oh, you’re stupid. You should have known that,’ then that’s not good. I think, if the doctor can see that they are causing the student a lot of angst and then they continue, that would make it abuse. I’m happy to talk about that because I feel like I get embarrassed really easily, but I feel like, for me, I don’t usually interpret being embarrassed as somebody else’s fault. Like, something that wasn’t done intentionally in kind of a mean way… but when someone humiliates you, it feels like they wanted you to feel embarrassed. They wanted you to feel bad, whether or not there was learning behind it.	Embarrassment – internally dependent from lack of knowledge Humiliation – purposefully induced embarrassment Abuse – intentional cause of harm
What other factors may play a role in one’s perception of public humiliation?	I would say we pick up the intent of the attending and whether their intent is positive, or there’s a … not necessarily malicious, but certainly a harsh edge, meant to cut you on a different level than your educational foundation. I think that will come through. It comes through in tone, a lot. And then I think one other time… that I thought looked really humiliating, was in front of a patient … it’s different when you say, ‘I’m going to do a little bit of teaching here, Sir. Do you mind if I teach while we’re kind of examining you?’ But if you’re shooting questions that students don’t know the answer to, it kind of undermines any tiny little bit of authority that we have, as medical trainees, and I think undermines any trust that the patient has in your knowledge base, if you’re getting questions wrong and looking really uncomfortable in front them.	Perceived intent of the teacher influences experience Negative treatment in front of patients and/or other staff – risk for humiliation
Where does ‘pimping’ fit in?	I definitely think that some people interpret Socratic teaching, in general, as something that is intended to single you out and make you feel uncomfortable, which I think can be interpreted as humiliating. But I would say that the same questions were asked of me, in probably a similar setting and my interpretation was that that was how they taught and that what they wanted you to get out of it was to learn the material and not to single you out and make you feel stupid. But when he was rude to me, in front of patients, it felt like, ‘You know, these are people I’m trying to build a rapport with and I’m trying to have them trust me to do exams and that kind of thing,’ that bothered me much, much more. I don’t know, I feel like pimping is kind of referred to as a negative thing, but I’ve found that it’s extremely valuable, if done correctly. I think setting expectations for the purpose of pimping is really, really helpful. Especially when attendings say, ‘I don’t expect you to know the answers to all of these questions …’	Perceived intent of the teacher influences experience Some enjoy and find the processes useful
How do these situations vary between specialties?	Not just the attendings (in reference to the OR). Sometimes people are really mean to you there. And not in a way that’s really that directive I would echo that I think the most negative interactions that I had, even though I don’t think I would really qualify them as mistreatment, were probably were kind of inter-professional, like with nursing or scrub techs in the O.R., I thought that those were probably the times where I felt kind of … that were most demeaning.	More difficulty with surgical specialties Negative interactions can come from all roles/professions
How does the Dean’s Office play a role in this problem?	… continual emails and continual touching in with students. And it’s very much on the table and above board. And I’ve found that trickles down, at least to the attendings That people I’ve talked to, at other schools, haven’t had that same experience and have had a lot more vicious environment that was really not as pleasant. It’s like, ‘I don’t want anybody to come out of this rotation having had a terrible experience. If you’re not having a good time, for whatever reason, let me know.’	Office felt to be very open Appears that the office is actively trying to improve things
What determines whether or not you report these incidences?	I think there are definitely situations where people probably perceive that they’re being either mistreated or humiliated, but I would be shocked if those people are actually reporting any of those things. I think, by the time that we get around to writing those feedback things, people just want to be done. It’s the last thing that they’re going to do, to think back to week two when that one person said that one thing to them. They do seem to be genuinely interested in having an open door and they’re a lot more receptive. So it does play a role in … I mean, so, if I had to go back, I probably would not report it just because I would be afraid of what could happen, if I saw that individual again and worked with them	Reporting can prolong a bad experience Influenced by anonymity and grading Institution is open to feedback
What needs to change to make this better?	Whereas, it’d be best if we could go straightaway, as it’s happening, and say, ‘Hey, this is happening. It’s not cool,’ and feel like we’re supported to the point that something would change, but it’s hard for the school. I think it would be great if anyone that did any type of clinical teaching had some sort of required teaching education. Just basic teaching tips – how to give feedback	More training for teachers on teaching skills Education on how to give feedback

Results from the quantitative survey given to participants at the beginning of each focus group are summarized in Table 3. Notably, the percentage of participants who reported experiencing ‘Public Humiliation’ (55%) was higher than reported from the 2015 AAMC Graduation Questionnaire (20%) [21]. Fifty-six percent of participants who reported experiencing public humiliation reported that they had experienced this ‘once’ vs 44% who reported that they experienced this ‘occasionally.’ Also of note, the study participants reported experiencing a wide variety of behaviors (not just public humiliation) defined as ‘mistreatment’ by the AAMC Graduation Questionnaire.

**Table 3.** Mistreatment survey results of study participants frequency the following behaviors were experienced by study participants, as indicated on their individual, quantitative, participant surveys. The first value in each column represents data from this study, the second, italicized value represents corresponding data from the 2015 AAMC graduation questionnaire [21].

	Never	Once	Occasionally	Frequently
Public Humiliation	45%/*80.5%*	31%/*10.3%*	24%/*8.6%*	0%/*0.6%*
Threatened with physical harm	97%/*98.4%*	3%/*1.2%*	0%/*0.3%*	0%/*0.1%*
Physically harmed	97%/*97.9%*	3%/*1.8%*	0%/*0.3%*	0%/*0%*
Required to perform personal services	97%/*92.1%*	3%/*5.1%*	0%/*2.6%*	0%/*0.2%*
Subjected to sexist remarks	62%/*85.9%*	10%/*5.9%*	28%/*7.6%*	0%/*0.6%*
Denied opportunities for training or rewards based solely on gender	86%/*93.6%*	0%/*2.7%*	10%/*3.2%*	3%/*0.4%*
Received lower evaluations or grades based solely on gender	93%/*93.8%*	0%/*4.0%*	7%/*1.9%*	0%/*0.3%*
Subjected to unwanted sexual advances	97%/*95.3%*	0%/*2.6%*	3%/*2%*	0%/*0.1%*
Asked to exchange sexual favors for grades or other rewards	100%/*99.8%*	0%/*0.1%*	0%/*0.1%*	0%/*0%*
Denied opportunities for training or rewards based solely on race or ethnicity	100%/*96.6%*	0%/*1.1%*	0%/*1.7%*	0%/*0.6%*
Received lower evaluations or grades solely because of race and ethnicity	97%/*97%*	3%/*1.5%*	0%/*1.2%*	0%/*0.3%*
Denied opportunities for training or rewards based solely on sexual orientation	100%/*99.5%*	0%/*0.2%*	0%/*0.3%*	0%/*0.1%*
Subjected to offensive remarks/names related to sexual orientation	86%/*97.6%*	10%/*0.9%*	3%/*1.1%*	0%/*0.1%*
Received lower evaluations or grades solely because of sexual orientation	100%/*99.6%*	0%/*0.2%*	0%/*0.1%*	0%/*0.1%*

## Defining ‘public humiliation’

The most consistent theme, that defined public humiliation, indicated that public humiliation is purposeful embarrassment caused by an outside party, with negative intent. Participants described that embarrassment is an internal state that is brought on by the individual being affected and is distinct from public humiliation. The following examples illustrate this further:

“I don’t care if I get something wrong. I’m wrong all the time. But if they make me feel like I’m an idiot for getting something wrong or if they make me feel like “Oh, you’re stupid. You should have known that,” then that’s not good.”

“I don’t usually interpret being embarrassed as somebody else’s fault. Like, something that wasn’t done intentionally in kind of a mean way… but when someone humiliates you, it feels like they wanted you to feel embarrassed.”

As an example to further illustrate this theme, if a student is asked a question and does not know the answer, he or she may feel embarrassed by this due to internal expectations that he or she should have known the answer. Making their lack of knowledge known is then embarrassing. In contrast, if the student perceives that person asking the question is doing so with negative intent (i.e., purposefully causing distress), then that experience would fall into the domain of public humiliation. A second theme illustrated that a situation becomes abusive when the perpetrator is perceived to know that his or her actions are causing the victim harm (i.e., distress and shame) and continues anyway.

“I think if the doctor can see that they are causing the student a lot of angst and then they continue, that would make it abuse.”

**Figure 1.**
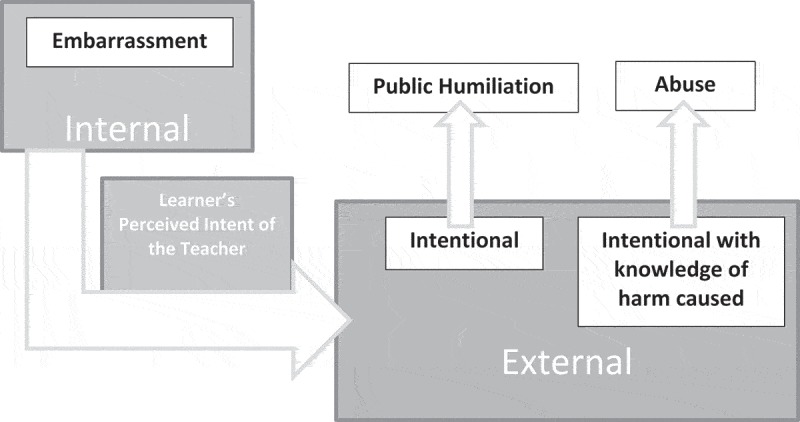
Experiences that can lead to public humiliation participants described that embarrassment is an internal experience that is self-created by behavior and/or lack of knowledge. Public humiliation can occur when there is a perception of an external participant intentionally causing the experience. Participants described that abuse (i.e., emotional abuse) can occur when there is a perception that an external participant intentionally caused the experience with knowledge of the fact that it is causing the learner harm.

These themes are illustrated in Figure 1.

## Risk factors for ‘public humiliation’

Several questions focused on identifying possible risk factors for public humiliation. The most consistent theme evident in the responses received was that perceived negative intent of a teacher could lead to a situation being considered to be publically humiliating. From participants:

“I would say we pick up the intent of the attending and whether their intent is positive, or there’s a … not necessarily malicious, but certainly a harsh edge, meant to cut you on a different level than your educational foundation.”

“And I feel like we can kind of know when somebody is being rude to you versus when somebody is just being hard on you.”

This theme was present in every focus group and is not surprising given that participants defined public humiliation as a forced experience of embarrassment, with negative intent. What was surprising was that participants in the group did not describe experiences of public humiliation in which a teacher purposefully created a situation where the learner would know that the teacher had negative intent. Instead, negative intent was assumed. Participants described that when a teacher-oriented the learner well, public humiliation did not occur. The following quote exemplifies this:

“And I think the times that pimping went the best for me, so to speak, is almost when it was preceded by attendings that would say, “Hey look, I’m trying to teach you and this is how I teach students is I ask them questions and you might feel attacked. That’s not what I intend to do.””

Additionally, participants described a theme that feedback directed towards personal characteristics, outside of medical knowledge or skill, is likely to induce perceptions of negative intent on the part of the teacher and the experience of humiliation.

“I think it becomes more humiliating than embarrassing, when they’re talking about personality things rather than academic things”

‘You know, if we’re talking about medicine, even if it comes off as harsh, then I won’t ever think of it as … well, not ever, but I would be less inclined to think it’s about something as mistreatment versus, if we’re talking about something that clearly has nothing to do with the reason I’m here’

Analysis also noted a theme that the setting in which an incident takes place is an important risk factor for public humiliation. Intuitively, more public (i.e., a greater number of observers) situations carry a greater risk of public humiliation. More specifically, public situations that involved patient care, in front of a patient, carried the greatest risk for the perception of public humiliation as exemplified by the following:

“But when he was rude to me, in front of patients, it felt like, “You know, these are people I’m trying to build a rapport with and I’m trying to have them trust me to do exams and that kind of thing,” that bothered me much, much more.”

“The attending critiqued her in the hall, in front of the nursing staff and stuff and … It just … it stung her and it stung me to watch because I knew how hard she had tried”

In addition to the type of observer present, participants cited that experiences of public humiliation appeared to be more common during medical procedures. Specifically, experiences in the operating room (OR) were commonly cited as examples of public humiliation.

## Factors not associated with ‘public humiliation’

Discussions during the focus groups of this study also delineated certain characteristics of training experiences that were not felt to be associated with public humiliation. Participants did not associate the role of the individual involved in an experience of public humiliation with a specific level of risk. Attending physicians, resident physicians, and nurses were all described as equally likely to be involved. Collectively, participants did not consider clinical rotations at either academic medical centers or community hospitals and clinics as more likely to be sites for public humiliation. The location of a clinical site (i.e., urban vs rural) was also not consistently linked to risk for public humiliation.

Participants were also clear to describe that ‘pimping’ in and of itself is not a risk factor for public humiliation. We should clarify that the term ‘pimping’ is not used because it is felt to be a correct description of Socratic questioning, but that it is the term the participants themselves used. It should also be clear that ‘pimping’ does not equate with a classic, Socratic technique, but more of a general teaching style in which questions are asked in a roughly Socratic manner. Focus group conversations on the topic of ‘pimping’ were particularly rich and delineated very clearly that participants could describe very positive experiences and very negative experiences that they often associated with public humiliation. The common theme between the two was the perception, on the part of the learner, as to the intent of the questioning. The questioning style itself was not at risk of leading to the perception of public humiliation if the learner felt that the questioning had positive intent and held the purpose of enhancing skill and/or increasing knowledge base. This was then an expansion on the theme of perceived negative intent from the teacher as being a risk factor for humiliation. The following examples illustrate this expanded theme:

“I definitely think that some people interpret Socratic teaching, in general, as something that is intended to single you out and make you feel uncomfortable, which I think can be interpreted as humiliating. But I would say that the same questions were asked of me, in probably a similar setting and my interpretation was that that was how they taught and that what they wanted you to get out of it was to learn the material and not to single you out and make you feel stupid.”

“I think there is some good to it. I mean, you want to … it’s a good way to learn anatomy and stuff, but I think there’s a point when it becomes you’re not even really trying to challenge them, you’re just trying to make them embarrassed.”

Participants described that if the learner was not oriented to the intent of the questioning, then it was more likely that the learner would assume negative intent, leading to the perception of public humiliation.

## Reporting of ‘public humiliation’ and ways to improve this experience

Though it was not a main focus of our investigation, we did ask questions relating to the reporting of experiences of public humiliation to medical school administration and how the clinical learning experience of students could be improved. Participants noted that they felt that students often would not report experiences of humiliation due to the fact that the experience had passed and they did not want to prolong it. In addition, their perception of anonymity and/or the possibility that reporting would affect their grade negatively impacted the likelihood that they would report an occurrence. Participants also noted that the medical school administration demonstrated openness to the reporting of mistreatment and efforts to limit this experience on the part of students. When asked about how to improve the educational environment of students and reduce experiences of humiliation, participants identified further instruction of teachers on teaching skills and feedback skills as being key recommendations.

## Discussion

Our study investigated the experience of ‘public humiliation’ among medical students at our institution. Because this study was limited to our institution and limited in size, we cannot claim that the findings generalize to all students everywhere. Our findings were supported by the literature, which we will illustrate in this section. We also feel that these findings provide useful, preliminary knowledge for larger, more robust studies.

Our study clearly defined the experience of ‘public humiliation’ among medical students on their clinical rotations as a created experience of embarrassment with negative intent on the part of the perpetrator. The study also highlighted multiple risk factors for the experience of ‘public humiliation’ with a clear focus on the perceived intent of the teacher. This finding is consistent with other studies that highlight the importance of communication between teacher and learner as integral to the valuation of a teaching experience [4,9]. In particular, Gran and Snell found that student perceived intent of a teacher is central to the student’s perception of a teaching environment [4].

The findings of our study are valuable in that many schools across the country are employing efforts to reduce or end medical student mistreatment. Some schools have focused their efforts on increasing awareness of mistreatment, offering routes for and encouraging the act of reporting mistreatment, counseling perpetrators, and/or commitments to ‘zero tolerance’ policies for the mistreatment of students [5,6,22,23]. While limited data exists on the efficacy of these policies, rates of mistreatment, as reported in the AAMC graduation questionnaire, are decreasing [13,21,23].

Mistreatment is far from eliminated, however, in that more than a quarter of graduating medical students still report experiencing the phenomenon [21]. Our study found one of the most significant risk factors for the experience of public humiliation was the perception that perpetrators of public humiliation have negative intent motivating their actions. Efforts to enhance teaching skills, particularly the orientation of students to the clinical learning environment and the teacher’s intent, could be effective in reducing the experience of public humiliation.

Our study also indicated that surgical and medical procedural experiences within medicine were a risk factor for public humiliation. This is not a new consideration; a study by Stone et al. hypothesized that this was due to the concern students have for making errors in the OR, which is, in itself, a high pressure/high stakes environment [24]. Our study was not large enough or designed to determine what might explain this finding. Interestingly, however, in the OR, involved parties (attending, resident, nurse, medical student) may not know each other well, the experience is frequently novel for the student, and there may be limited time for orientation. Those factors work against optimal orientation to the learning environment and the intent of teaching interventions. Future studies could investigate this further to better understand the factors that led to this finding.

Our study sought to define and differentiate public humiliation, but the overlap with the experience of embarrassment on the part of the student is difficult to deny. While our participants delineated the difference clearly, it is very possible that the average student does not make such a clear distinction in the moment of such an occurrence. Highlighting this further, the AAMC graduation questionnaire added ‘embarrassment’ as an occurrence within the umbrella of behaviors associated with medical student mistreatment in 2013 [21]. With the addition of this item, ‘embarrassment’ has become the most frequent occurrence of medical student mistreatment with ‘public humiliation’ running a close second [21]. We suspect that the distinction between the two, that our focus groups illuminated, holds true and that the perceived intent of the perpetrator is central to an event being perceived as humiliating versus embarrassing. We would also postulate that our participants were correct in that embarrassment is, in fact, an artifact of the individual holding the emotion, while humiliation is differentiated by the perpetrator’s intent.

While not a central finding of our study, our analysis also indicated that students are less likely to report episodes of public humiliation or other forms of mistreatment if they perceive that it will negatively impact their grade or prolong the negative experience. This is consistent with the literature on this topic as fear of reprisal and a desire not to undertake the time and effort involved in reporting have been identified as reasons that limit reporting of medical student mistreatment [25]. Participants also recommended that continued education on teaching skills and feedback skills would improve the learning environment; a continued reminder that education and training on these skills should not be undervalued. Additionally, the perception of negative intent on the part of a teacher could, at times, be a reflection of a learner’s lack of orientation to the learning process or the experience of feedback itself. Education on receiving feedback as a learner may then also help improve the learning environment by orienting learners to this aspect of the teaching experience and limiting assumptions about novel processes.

Our study has a number of limitations. The most significant of these are the small size of the study and a selection bias in the medical students who were willing to participate in a focus group discussing mistreatment. Our study may well then not be reflective of medical students as a whole in our institution and/or medical students across the country. This study also did not look at the experiences or attitudes of attending physicians in similar situations and thus was limited in its perspective. In addition, participants may have felt inhibited in their responses during the focus groups as the moderators were study investigators in the department of the rotation the students were starting that day. Participants may have felt the need to modify their answers because of that aspect of the environment. We minimized this effect through the fact that none of the moderators had any supervisory capacity over any of the participants and did not work at the physical site of the student’s rotation, thus not coming into contact with the study participants outside the study. Our focus groups may also have been limited in scope and may not have investigated the topics in sufficient depth to illuminate more subtle aspects of ‘public humiliation’ and medical student mistreatment. However, responses were consistent across groups and time, and rapidly reached saturation. We also suspect that our findings are not unique to our institution.

## Conclusion

Our study is the first qualitative focus group study to specifically investigate student perceptions of ‘public humiliation’ in clinical medical education. The study was small and limited in its conclusions, though it provides areas for further study to better understand this important topic. While some of the conclusions we were able to draw may appear self-evident (i.e., orientation of learners to the teaching experience is not a novel concept) they remind teachers of the value of these practices, which may not be used as frequently as would benefit students. Larger, more robust studies should be undertaken to further investigate these preliminary findings.
